# Supplementary material for: Patterns of Intron Gain and Loss in Fungi
Source: PLoS Biol. 2004 Nov 30;2(12):e422. doi: 10.1371/journal.pbio.0020422 (PMC532390; doi:10.1371/journal.pbio.0020422)
Supplement: Table S1 — Also available at http://genes.mit.edu/NielsenEtAl/. (4.3 MB ZIP). [file pbio.0020422.st001.zip › NielsenEtAl/html/989.html]

AN6269.1.NCU06333.1.MG06172.1.FG06177.1


```
 CLUSTAL W (1.82) Multiple Sequence Alignments - Introns Inserted


Sequence 1: NCU06333.1	405 aa
Sequence 2: MG06172.1	382 aa
Sequence 3: FG06177.1	391 aa
Sequence 4: AN6269.1	394 aa
Alignment Length: 413 aa
Number Identitical Residues: 159 aa
Alignment Score (without introns) 8633


MG06172.1 	MSE------PQQGQ----MPPGMVRP--TPEQIAAMQR-QIAADAERAGMTVPEFIEHIK
NCU06333.1	MEQGP----PQPGPGQMPMPPPGQMP--TPEQIQAIQR-QLAIDAEKAGMTVPEFVEQLK
FG06177.1 	MSAPPPQPGPQPGPGQIPMPQPGQRP--TPEQIQAMQR-QLAIDAEKNGMTVPEFIEHIK
AN6269.1  	MATPGQPTPQQLAAMQQQFAAEAAKRGLTPEEFAKQQREQLNAEAAKHGMTTEQYVTQLR
          	*      .  * .  .  :.      . ***::   **.*:  :* : ***. ::: :::

MG06172.1 	KQ-QMEMMRQQQQQQQQQQQG-------HEGHDNDDDEDHGHQQ-QQEGQGQPITPGPPN
NCU06333.1	RQHQQMMMRQMQMQQQQQQQGGGGGPPPEDGHQHQHQHQHQHQHGPPAGQARPIVPGPPK
FG06177.1 	RQ-AQEQMRMRAQQQQGGDHGHG----HDHDHDHDHSHPHPHQQ-QQQGRSQPITPGPPN
AN6269.1  	MRAMAAQQKAAEVQRQLQANG------QSQGPPRPGQPGQPGQP-QTHTQQVPVNPNSPK
          	 :      :    *:*   :*        ..  .  .  :  *      :  *: *..*:

MG06172.1 	-PLAIALANFLKSQDLKPRTCILNGERKDMFRV1KRALRAIQSPAYEKARKKNPALPEVT
NCU06333.1	-PEAIALAKFLRSQELKPRTVILNGERKDMFRV1KRALRALQSPAYEKARKKNPLLPEIT
FG06177.1 	-PKALALAAFLRGQDLKPRTVIMNGERKDMFRV1KRALRALQSPAYEKARKKNPLLPEIT
AN6269.1  	DPKAIAVAQFLRSQNLKPRTCIMDGQRKDMFKV1KRAIRALESPAYEKARKKNPLLPPVT
          	.* *:*:* **:.*:***** *::*:*****:* ***:**::************ ** :*

MG06172.1 	DRASLENCFKLLPLSMLALRVNKVDPHEGHDHPPKK-----GKRVKGLWTVRIEPQQEAG
NCU06333.1	DRASLENTFKLLPMSMLALRVTKIDPHEGHDHAPTKKPHGGGKRVKGLWTVRIEPQQDAQ
FG06177.1 	DRASLENTFKLLPLSMLALRVSKIEPQAGPNGKKPK-------RVKGQWTVKVEPQQEAQ
AN6269.1  	DRASAENVFKLLPLSVLALRVSKVDPHAGHGHGKTK-------RVKGLWTVKIEQHQEAD
          	**** ** *****:*:*****.*::*: * .    *       **** ***::* :*:* 

MG06172.1 	DDMYYVWLYEGSQIMRKVYAALALVVIFAIVLYPLWPLFMRQGVYYLSWGFLMLLGLFFL
NCU06333.1	DDMYYVWLWEGSQVMRKVYAALALLVIFAVVLYPLWPLKMRQGVYYLSWGFLMFLGLFFA
FG06177.1 	DDMYYAWLWEGSQLKRKIYAGLALLAIFAVVLYPLWPLVLRQGVYYLSWGLLGLLGLFFL
AN6269.1  	PMMHYVWLYEGPQWKQKAMAAAVVAGIFAVVLFPLWPIMLRQAVWYLSVGMMGLLGLFFA
          	  *:*.**:**.*  :*  *. .:  ***:**:****: :**.*:*** *:: :***** 

MG06172.1 	MAIFRVILFCVTYFVLSPGLWLFPNLWEDVSFVDSFKPVWAWHE0TEKKKKKKSKAGTSG
NCU06333.1	MAIFRVILFCITYFVLSPGFWLFPNLWEDVSVVESFKPVWAWHD0PNPKKKKKNKSKTPA
FG06177.1 	MAIFRVILFCITYFTNSPGLWLFPNLWEDVSFMDSFRPVWAWHE0TEKKKKKKKSTVAVP
AN6269.1  	MAIFRLILFCITVFAVPPGLWLFPNLFEDVGFFDSFKPLWGWQE0TGKKKKKSKKAASSA
          	*****:****:* *. .**:******:***...:**:*:*.*:: .  ****...: :  

MG06172.1 	GSSKAGAAFAGATGQPLPASATTTATETMVATSP---VERRGYTAPQVEELDDDE
NCU06333.1	GSAKANATFAATTGQPAPATAVTTATDTQVATATPTAAAQRNYVAPKVEELADDE
FG06177.1 	GTS-VNPALAGATG-LAPTSATTTGTDTQVKTGD---VQQRHYEAPRVEELEDDA
AN6269.1  	AQTSNPQATESAPAATAETAPAATTTATESADAAG---PVKRDLAPRVEEVEE--
          	. :.   :  .:..    ::..:* * *    .       :   **:***: :
```
